# Multifactorial Patterns of Low Performance Development in German Elite Athletes

**DOI:** 10.1155/tsm2/8421509

**Published:** 2025-10-05

**Authors:** Kati Wiedenbrüg, Andrea Roffler, Lukas Reichert, Michael Mutz, Karen Zentgraf, Karsten Krüger

**Affiliations:** ^1^Institute of Philosophy II, Martin-Luther-University Halle-Wittenberg, Halle 06210, Germany; ^2^Institute of Sport Sciences, Goethe University Frankfurt, Frankfurt am Main 60487, Germany; ^3^Institute of Sport Science, Justus-Liebig University Giessen, Giessen 35394, Germany

**Keywords:** lower body dynamics, mental well-being, metabolism, nonergodicity, social support

## Abstract

**Introduction:**

Low performance development (LPD) has been related to several training-related, biological or psychosocial factors. However, there is still hardly any comprehensive research on its multifactorial nature. This study explored whether factors previously associated with LPD manifest as cross-disciplinary pattern combinations across such athletes.

**Methods:**

Cluster analyses were computed based on performance-related (speed and strength of the lower body), biological (ratio TNFα:IL10; fT3, leptin, insulin) and psychosocial (perceived social support; mental well-being) data from 62 of 296 elite athletes whose performance development was below the samples' average range. Group comparisons were calculated for demographic, anthropometric, nutritional and sleep-related variables, as well as for additional psychosocial (critical life events; perceived stress) and biological (single inflammatory markers) variables.

**Results:**

Six patterns were identified, which could be described via domain-specific characteristics (lower body dynamics and social support), via an interdisciplinary combination of factors (social support, mental well-being and/or lower body dynamics) or by no characteristic pattern at group level.

**Conclusion:**

In general, this study extends research on LPD and illustrates the limited validity of generalisations while emphasising the additional value of individualisation for athletes who drop behind their peers. Moreover, the identified patterns point to the limitations of taking a cross-disciplinary approach at group level.

## 1. Introduction

Elite sport is a highly competitive environment in which performance substantially influences the athletic career from junior-to-senior transition [1, 2] until retirement [3, 4]. On the way to the top, practice and the process of performance development (PD) are decisive components [5–7], and both are also closely interlinked to adaptation, load capacity, health and hence further performance [8]. Conversely, it is of serious concern when athletic performance does not align with personal objectives, the training regimen undertaken and the expectations set by coaches, because perceived performance stagnation or decline can potentially compromise an athlete's career prospects [3, 9]. Moreover, an athlete's health can be threatened by, for example, poorly tolerated training loads, an imbalance between training and recovery or psychological stress [8, 10, 11].

**Figure 1:**
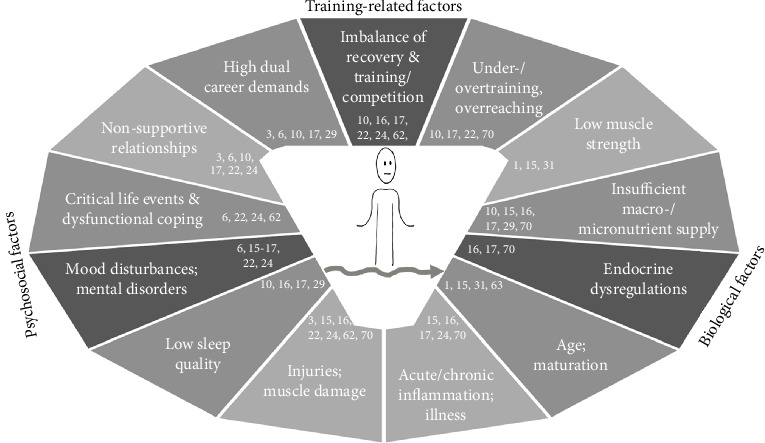
Factors possibly contributing to low performance development.

It is widely agreed that PD is multifactorial. Personal and environmental characteristics of a biological, psychological and social nature interplay in each athlete in a unique, complex and dynamic way, affecting physiological and psychological reaction and adaptation to training [11–13].  Figure 1 presents an overview of factors associated specifically with low performance development (LPD). One of these biological factors is an insufficient macro- or micronutrient supply. The bodily and physiological adaptations triggered by an insufficient nutrient supply can affect the energy metabolism as well as the immune, endocrine or muscle functions, and these, in turn, can acutely and chronically impair health and performance [14–16]. When macronutrient deficiency persists over time, health- or performance-related symptoms such as metabolic dysregulations, menstrual irregularities or increased susceptibility to injuries can manifest. This cluster of symptoms caused by LEA has been subsumed under the sometimes criticised syndrome “Relative Energy Deficiency in Sport” (REDs) [17, 18]. One of the psychological factors depicted in Figure 1 is mood disturbances and mental disorders. Although there have been previous reports on a concurrence of low mental well-being, mood disturbances or mental disorders with LPD [11, 18–20], mental well-being is generally not seen as a necessary condition for athletic performance [21, 22]. Instead, it is seen as a resource increasing the chance that performance will be good in the long term [7, 21, 22]. Exemplary for the social factors, which can but do not necessarily have to hamper performance and its development are, for instance, difficult or non-supportive relationships [7, 23, 24]. Here, an influence on PD is explained through the lack of emotional and instrumental support [7, 25, 26] or through its relation to the stress that can be evoked or buffered in an athlete [27, 28].

Although the factors in Figure 1 are depicted separately, it is important to consider that none of them function in isolation. Instead, their multifactorial interaction affects LPD etiologically, which is precisely why the present investigation of the cross-disciplinary combination of multiple factors extends research on LPD [11, 13, 18, 29]. Yet, its multifactorial nature has not been addressed adequately in previous studies. Some have examined factors solely from a single research discipline, as is the case in research approaching LPD from a biological [1, 30, 31] or psychosocial perspective [3, 23]. Other studies have regarded multiple factors in a cross-disciplinary way, as in the case of REDs, but have been criticised for vague concept definitions (for critical reviews, see, e.g., Jeukendrup et al. [18] or Weakly et al. [10]). Consequently, it is not possible to conclude whether the factors already associated with LPD manifest themselves as specific pattern combinations across athletes whose PD is low.

However, knowledge about recurrent patterns across elite athletes with LPD would be beneficial for research, practice and the athletes themselves. The identification of patterns and the formation of corresponding subgroups would help to organise and understand multivariate data, while maintaining both the depth of detail at the individual level and the comprehensiveness at the construct level [32, 33]. This again would ease communication to and between practitioners and researchers [32, 33]. Moreover, knowledge about patterns across athletes would serve to customise support [34, 35] and facilitate personal intervention planning to enhance, for example, athletic performance [36, 37] and/or mental well-being [38].

Therefore, the aim of this study is to explore whether biological and psychosocial factors manifest in specific patterns across athletes with LPD in ways that would enable a formation of distinguishable subgroups of elite athletes with similar patterns.

## 2. Materials and Methods

This study was conducted within the collaborative, multidisciplinary research project ‘Individualized performance development in elite sports through holistic and transdisciplinary process optimization' (*in:prove*). The study protocol is in accordance with the Declaration of Helsinki and was approved by the university ethics committee (number: AZ 55/22). Prior to the study, each athlete or their legal guardian was informed in written and oral form about data acquisition, data handling and data privacy before signing a declaration of consent. Consenting only to some specific measurements was possible. Following the given written consent, an interdisciplinary research team collected demographic, anthropometric, performance-related, psychosocial and physiological data in permuted order while athletes attended training camps for the junior or senior national teams. The research team included a medical professional to collect blood samples. In addition, athletes were asked to provide nutritional and sleep-related data.

### 2.1. Participants

Given that the aim of the study was to investigate LPD in elite athletes, sample selection was based on the athletes' PD as evaluated by the respective national coach. This operationalisation through coaches' assessments was chosen, because these are generally considered to be prognostically valid and to process extensive implicit and explicit knowledge about the athlete holistically [39–41]. Concretely, PD over the last year was evaluated on a seven-point Likert scale (1 = no development or decline to 7 = strong development).

From a cohort of 296 elite athletes (for further information, see Zentgraf et al. [42]), a sample was selected whose PD was identified as low. The selection criterion followed the convention in diagnostic psychology (see Marburg conventions [43]), as the PD followed a unimodal distribution, which was only slightly skewed (−0.61, SE = 0.14) and had a kurtosis of −0.02 (SE = 0.28; for more details, see Supporting Figure A). Accordingly, low was defined as underscoring the average range (*M* ± ½ SD). Based on the values of the 296 athletes, this resulted in a cut-off value of PD ≤ 4.07. Thus, the sample in which patterns were to be identified encompassed 110 elite athletes (70 female; *M*_age_ = 18.85 ± 4.63 years) from the disciplines artistic gymnastics, 3 × 3 basketball, ice hockey, modern pentathlon, rhythmic gymnastics, trampoline gymnastics, table tennis and volleyball. All athletes were members of the national junior or senior team.

### 2.2. Demographic, Anthropometric and Performance-Related Parameters

Demographic details of the participants (sex, age, athletic discipline) were passed to the research team in advance. On the day of measurement, the athlete's height (in cm) and weight (in kg) were measured, and the body mass index (BMI) was calculated. Athletes were asked to report any acute injury (no/yes). All athletes provided information on when they started practicing their main sport, which was used to calculate years of practice. Female athletes were interviewed about their menstrual or hormonal cycle (regularity of cycle, cycle and bleeding duration, use of contraceptives, cycle-related symptoms) in a confidential 5–20-min conversation (separate room). According to the IOC statement paper by Mountjoy et al. [17], a natural menstrual cycle was defined as regular with 35 days or less between the periods for at least 8 periods per year.

Lower-body dynamics were estimated by discipline-specific power- and speed-related components of the lower extremities, including jump performance and tapping or sprint performance, respectively. The respective performance tests were selected based on the current literature (such as Gaspari et al. [44], Pelletier et al. [45] or Wen et al. [46]) and in consultation with the national coaches. Jump performance (jump height in cm; Microgate, Bolzano, Italy) was measured by countermovement jumps (CMJ; 3 × 3 basketball, artistic gymnastics, ice hockey, trampoline gymnastics, volleyball), drop jumps (DJ; rhythmic gymnastics, table tennis) or sergeant jumps (SJ; modern pentathlon). The SJ were measured with a jump-and-reach lab-built device. Performance in SJ was relativised by each athlete's height of reach in standing position. Tapping performance encompasses the maximal tapped frequency (in Hz; Voss, Doberschütz, Germany) within 5 s and was gathered in the disciplines 3 × 3 basketball, artistic gymnastics, modern pentathlon, rhythmic gymnastics, trampoline gymnastics, table tennis and volleyball. Sprint performance encompasses the time (in seconds; Microgate, Bolzano, Italy) athletes needed to sprint over 10 m from a frontal starting position and was gathered in the discipline ice hockey. To calculate total scores, first the sex-specific *z* standardised values were calculated across the 296 elite athletes separately for each performance test. Subsequently, these *z* transformed values of the jump and tapping (artistic gymnastics, 3 × 3 basketball, modern pentathlon, rhythmic gymnastics, table tennis, trampoline gymnastics, volleyball) or jump and sprint performance (ice hockey) were averaged to the total score.

As a further performance-related parameter, the respective national coach assessed the athlete's performance level over the last year on a seven-point Likert scale (1 = very low to 7 = very high).

### 2.3. Psychosocial Parameters

All psychosocial parameters were collected by a questionnaire that athletes filled out either by paper and pencil or online, depending on their athletic schedules on measurement days. To screen for mental disorders, the questionnaire contained the Patient Health Questionnaire-4 (PHQ-4) by Kroenke and Spitzer [47]. This is a four-item self-report screening tool investigating the occurrence of depressive and anxious symptoms over the last 4 weeks (two items each) on a four-point Likert scale (1 = never to 4 = nearly every day). A total score (mental well-being) was calculated by averaging and inverting the four items, with a lower score indicating more depressive or anxious symptoms.

The (lack of) social support was assessed with the Multidimensional Scale of Perceived Social Support (MSPSS) by Ziemet et al. [48]. This contains 12 statements on perceived general support from different social subgroups (friends, family, significant others) that are each rated on five-point Likert scales (1 = strongly disagree to 5 = strongly agree). A total score (social support) was calculated by averaging the MSPSS items with a lower score indicating a lack of social support.

Subjective stress was surveyed by adding the four-item Perceived Stress Scale 4 (PSS-4) by Cohen et al. [49] to the questionnaire. Athletes used a five-point Likert scale (1 = almost never to 5 = almost always) to rate how far these statements applied to them over the last month. A total score (perceived stress) was calculated by averaging the four items, with a higher score indicating higher perceived stress.

Thirteen yes/no items were used to measure the presence of different critical life events (CLEs) such as major social conflicts, injury or death over the last year. If an event occurred, the burden it imposed was rated on a five-point Likert scale (1 = very low to 5 = very high). For each athlete, the occurrence of CLEs with a subjective burden > 3 was counted and summed up as a total CLEs score (maximum: 13), with a higher score indicating the experience of a greater number of high-burden CLEs during the last year.

### 2.4. Physiological Parameters

All blood sampling was conducted before noon. To minimise the inference of the measurement with the daily and training routine as well as with other measurements taken (e.g., performance tests), the elite athletes came in a fed state. They usually did not train before blood sampling on the measurement day, which resulted in a minimum time gap of 10 hours between last training and blood collection.

Different physiological parameters were extracted from venous blood (about 25 mL). Blood samples were collected using 7.5-mL Serum-Gel CAT Monovettes (Sarstedt). After venipuncture, the samples were allowed to clot for approximately 30 min at room temperature. They were then centrifuged at 2000–3000 × g for 10 min at 4°C. The resulting serum was aliquoted into sterile microtubes and immediately stored at −80°C until analysis. No freeze–thaw cycles occurred prior to measurement.

The Luminex (LX)-200 instrument from the Luminex Multiplex Assay Kit was used to assess concentrations (each in pg/mL) of inflammatory parameters such as interferon gamma (IFN-γ), tumour necrosis factor alpha (TNF-α) and the interleukins (IL) IL-1β, IL-6 and IL-10, as well as the levels of the insulin-like growth factor binding protein 1 (IGFBP-1), the hormones insulin and leptin. Using immunoturbidimetric analysis, the concentration of the C-reactive protein (CRP; in mg/dL) was evaluated. A chemiluminescent immunoassay (CLIA) was used to analyse the hormone level of free triiodothyronine (fT3; pmol/L), as well as the concentrations of ferritin (in ng/mL), vitamin B12, vitamin B9 and 25-OH-vitamin D (both in ng/mL). Using a cell counter (Sysmex Hematology Analyzer, Norderstedt, Germany), a complete blood count (CBC; in 10^9^/L) was conducted from EDTA blood.

A total score for metabolism was calculated by averaging the *z* scores of fT3, insulin and leptin, which were standardised previously across all 296 athletes. Moreover, the ratio TNF-α:IL-10 as well as the neutrophils-to-lymphocytes ratio (N:L ratio) was calculated to operationalise the inflammatory response through a single variable. Here, higher ratios indicate a more pronounced, and lower ratios a more balanced inflammatory condition.

### 2.5. Nutritional and Sleep Parameters

Nutritional and sleep data were self-assessed after the measurement day for three typical days (two weekdays and one weekend day) with a nutrition protocol and a sleep protocol. Protocols were then sent back to the research team.

The nutrition protocol was semistructured. For each protocol day, athletes received one table, where the quantity of all food and liquids consumed should be documented in the respective column (breakfast, lunch, dinner, and snacks) and row (food and drinks) per paper and pencil. Prior to handling out the protocols, athletes were briefed about the importance of an accurate documentation and informed that quantities in terms of “a cup, a plate, a small/large portion, a tablespoon”, etc. are less accurate than weighed amounts, which yet also were accepted. On the protocol, athletes could also report any further supplementation or medicament intake including the dosage and name of the products. The returned protocols were transferred to the software DGExpert Version 2.0.45.1 (DGE, Bonn, Germany), which was used to evaluate the relative intake of macronutrients (carbohydrates, fat, proteins) and the total daily calories. Considering body size, age, sex and the physical activity level (PAL value = 2.0), the software calculated the relative intake (in %), which indicates the extent to which the real intake matches the reference values recommended by the German Nutrition Society (DGE).

The sleep protocol noted the time of going to beds, the sleep duration as well as sleep quality ratings (1 = very good to 5 = bad) per protocol day. These were used to calculate the average sleep duration (in hours) and average sleep quality.

### 2.6. Statistical Analysis

All statistical analyses were performed using IBM SPSS Statistics for Windows Version 29.0.2.0 (IBM Corp., Armonk, NY, USA) and the integrated R plugin (R Version 4.4.1; R Core Team, R Foundation for Statistical Computing, Vienna, Austria). To gain an understanding of the data structure, descriptive (missing cases, minimum, maximum, mean, standard deviation, skewness) and correlative (Spearman's Rho) pre-analyses were calculated for each variable. Next, patterns across the athletes with LPD were analysed through cluster analyses (CAs). Variable selection was guided by theoretical relevance and the independence of variables from each other. This was checked in a correlative pre-analysis (see Supporting Table A). Accordingly, the CA were based on the following variables: the PHQ-4 and the MSPSS representing psychosocial variables, the lower body dynamics score representing performance-related variables and the ratio TNF-α:IL-10 and the metabolism score representing physiological variables (see Table 1). If not performed previously during scoring, all variables were *z* standardised (across the 296 athletes of the total sample) to unify the scales. In all CA, cases with missing values were excluded list-wise, leading to an exclusion of 47 athletes with incomplete datasets due to consenting only to some selected measurements. To identify outlying cases, which can possibly distort especially the centre of a cluster [50, 51], the first CA was calculated using the single-linkage procedure (distance measure: squared Euclidean distance). Hence, one outlier (female; injured; metabolism score saliently far above average range) was excluded from further clustering. Then, another hierarchical CA (Ward's method; squared Euclidean distance) was performed across the remaining 62 cases to ascertain the number of distinct clusters in a data-driven way. Subsequently, a *k*-means CA was conducted to optimise the clusters. The number of clusters was set according to the previously determined number, and the maximal number of iterations to 10. To estimate the stability of the final cluster solution, a resampling method (bootstrapping with 1000 iterations) was computed using the R-packages ‘boot' version 1.3–30 [52, 53] and ‘mclust' version 6.1.1 [54]. In each iteration, 62 random samples were drawn from the original data set and the k-means algorithm was executed. By projecting all generated cluster centres back to the original data, finally the adjusted rand index (ARI) was calculated for each iteration, which were then averaged to a total ARI.

To describe the clusters, primarily the cluster centres were examined, with a characteristic deemed relevant, if the cluster centre undercut *z* = −0.5 or exceeded *z* = 0.5 and therefore lied outside average range according to the Marburg conventions [43]. To complement cluster description, descriptive statistics and non-parametric mean comparisons for independent samples (Kruskal–Wallis tests) were carried out using parameters, which had not been used for clustering before (for an overview see Table 1). Here, a feature was considered as characteristic, when one subgroup significantly (*p* ≤ 0.05) differed to the others in the post hoc group comparisons. Missing values were excluded case-wise test by test. To ease reading, the mental well-being and the inflammatory balance scores were used for cluster description. These are the inverted PHQ-4 and the inverted ratio of TNF-α:IL-10. The descriptive wording also followed the Marburg conventions [43]: according to an average range of M ± ½ SD, *z* values from −0.5 to 0.5 were defined as “average”, *z* values exceeding 1.0 and 2.0 were defined as “high” and “very high,” respectively; analogously, the descriptions “low” and “very low” were used for values *z* < −1.0 or *z *< −2.0. Generally, the level for statistical significance was set to *α* = 0.05.

## 3. Results

In total, 62 out of the 110 elite athletes with LPD were included in the CAs as they neither had missing nor outlying data in the variables used for clustering. Detailed participant characteristics for this sample can be found in Table 2.

The dendrogram of hierarchical clustering based on Ward's method (see Supporting Figure B) suggested a three-, four- or six-cluster result. To minimise heterogeneity within a subgroup, it was decided for the six-cluster solution. Details about the subgroup characteristics for the three- and four-cluster solution are provided in the Supporting Table B.

**Figure 2:**
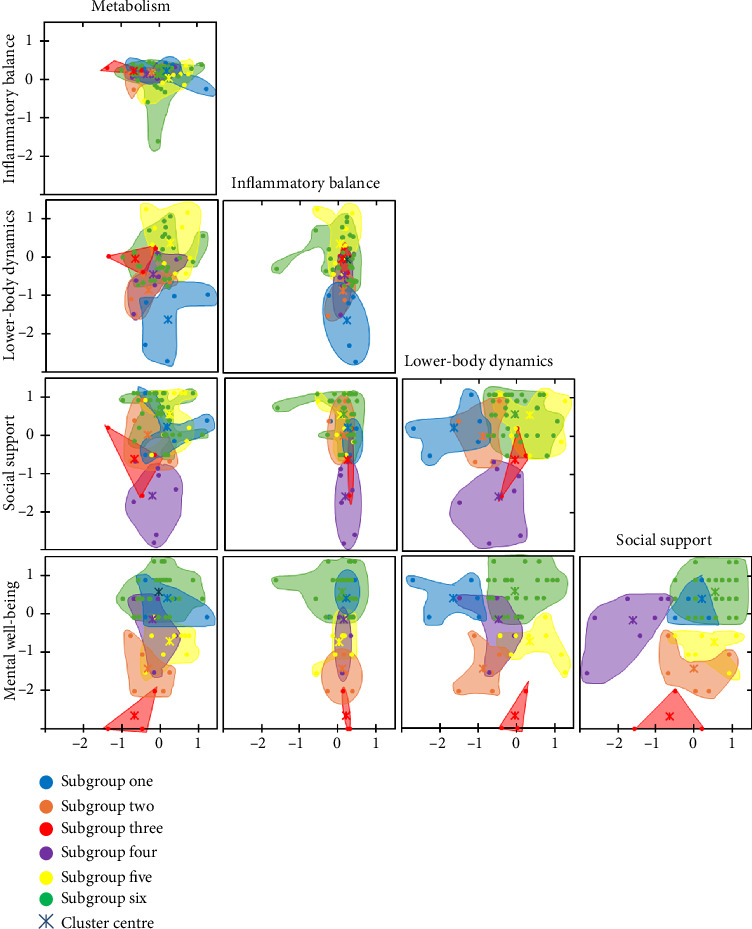
Graphic representation of the identified patterns based on the variables used for clustering.

According to the six-cluster solution (silhouette score = 0.11), the patterns of the subgroups were differentiated by single, domain-specific characteristics (subgroup one and two), the combination of interdisciplinary characteristics (subgroups three to five) or the absence of characteristics at subgroup level (subgroup six). The respective characteristics are highlighted in bold in Table 3, which contains detailed, subgroup-specific descriptive statistics for each variable used for clustering. Each elite athletes' variable values alongside their respective subgroup affiliations are visualised in Figure 2. It depicts that the factor of inflammatory balance does not discriminate well between the subgroup patterns. For the factor of metabolism, subgroup three (red) is slightly accentuated attributable to its left shift (first row). In the second row of Figure 2, where the factor of lower body dynamics is plotted, subgroup one (blue) stands out by its downward shift reflecting their characteristic of very low lower body dynamics. Similarly, the subgroup three (red), four (purple) and five (yellow) protrude regarding social support, and subgroup two (orange), three (red) and five (yellow) regarding mental well-being. For subgroup six (green) often a large patch located in the upper middle can be noted in Figure 2, which on the one hand reflects a certain variance within the subgroup and on the other hand indicates that at subgroup level no variable used for clustering deviates greatly to the positive or the negative.

This larger subgroup (six) had no characteristic pattern at the group level but the highest ranges in all variables except for the number of burdensome CLEs, the concentration in CRP and the number of symptoms during the menstrual cycle (see Supporting Table C).

To facilitate interpretation, additional demographic, physiological, psychosocial, nutritional or sleep-related variables were employed to describe the patterns (Table 3). Here, only the perceived stress (*H* [5] = 14.12, *p*=0.015) differed significantly between the subgroups, but pairwise post hoc comparisons exceeded the significance level of *α* = 0.05 after Bonferroni correction. All other variables such as age, discipline, the concentrations of specific inflammatory markers, the amount of CLEs, menstrual characteristics, macronutrient intake or sleep did not differ statistically significantly between subgroups (see Supporting Table C). However, nutritional and sleep information was only available for 0%–69% of the athletes in the particular subgroup.

The cluster stability was low for both the six- (ARI = 0.28; 0.06–0.83) and three-cluster solutions (ARI = 0.31; 0.03–1.00). The three-cluster solution (silhouette score = 0.12) reflected the patterning relevance of the lower body dynamics, mental well-being and social support. More precisely, one subgroup was characterised via a low score in lower body dynamics and mental well-being above average range, the second via low social support and low mental well-being, and analogue to subgroup six, no characteristic at subgroup level became apparent for the third subgroup (for more details, see Supporting Table B).

## 4. Discussion

The aim of the present study was to explore whether biological and psychosocial factors manifest in specific patterns across athletes with LPD. Of the six patterns identified, two were characterised via a single distinctive variable, three via a cross-disciplinary combination of factors and one pattern did not have any outstanding characteristics at the group level. A pattern such as the latter also became apparent in the three-cluster solution, alongside a pattern demonstrating a combination of biological and psychosocial characteristics, and a pattern characterised exclusively by psychosocial variables.

In sports science, it is often assumed that a multidisciplinary approach provides additional insights. In our case, three out of six clusters combined data from sports science sub-disciplines. It was only in these three clusters that the expectation of finding links between biological, training and psychosocial parameters proved accurate. Hence, through its data-driven and exploratory approach, this study confirms a certain relevance of biological and psychosocial factors as well as their concomitant manifestation for LPD. At the same time, it must be recognised that this complex, combined view of the data is also limited. At least at the group level, it resulted in unstable cluster solutions and identified a large group of athletes with no significant, noticeable characteristics. This again simultaneously illustrated the so-called ergodicity problem [42, 55] in the context of LPD.

Generally, the interpretation of some patterns can be well integrated into previous findings. For instance, for two subgroups in the six-cluster solution, the biological factor lower body dynamics was of relevance, which combines strength- and speed-related components of the lower extremities. Considering that sprinting and jumping are highly relevant for performance in the disciplines investigated [42, 56], it seems plausible that a deficiency in precisely these physical abilities is the central aspect for LPD in the first subgroup. In the other subgroup (subgroup two), however, it is reasonable that it is not the low lower body dynamics per se but rather the injuries that hinder PD and good mental well-being. Findings that injured athletes are less likely to progress in their discipline [57, 58] and more likely to be exposed to a risk factor for mental well-being [20, 59] support the suggested rationale for the second subgroup. To enhance PD and long-term success, both subgroups may benefit particularly from interventions aiming to increase strength and/or speed or to prevent injury [60, 61]. Notably, the present study reveals no significant age difference between all subgroups although the relationship between muscle strength, performance, injury and maturation is assigned a special role especially during puberty [31, 62, 63]. Because the age distribution especially in the first subgroup is heterogeneous and encompasses an age range of about 14 years, and because this study does not assess maturity measures, no conclusions can be drawn on whether the degree of maturation influences the speed- and strength-related performance or the incidence of injuries. Nevertheless, the identification of two patterns with characteristic low lower body dynamics underlines the importance of both the strength- and speed-related components for developing performance.

Another pattern (subgroup three) in which a biological factor contributes to characterisation showed several saliences that overlap with the symptom cluster associated with problematic LEA or the possibly resulting REDs: First and foremost, stand the abnormalities in metabolism, indicated by the frequently reported low concentrations of fT3, leptin or insulin [16, 17, 64]. Very low mental well-being is another characteristic of this subgroup, which also fits to previously reported mental health issues [16, 17]. The high values in single inflammatory markers such as TNF-α and IFN-γ, the 66.7% prevalence of injuries and the lowest average performance level overlap with further symptoms of energy deficiency such as unbalanced immunity, an increased susceptibility to injuries and impaired athletic performance [16, 17, 65]. Despite this multitude of overlapping saliences, an LEA cannot appropriately be attributed to the athletes of this subgroup, mainly because no information was available on the caloric intake of the assigned athletes. Subsequently, their energy intake could not be estimated, which indeed would have been indispensable to verify an energy deficiency or LEA [16, 17]. At this point, it should be noted that the non-return of the nutrition protocols by one entire subgroup can have many causes, one of which may be to disguise unhealthy eating behaviour. However, this can neither be ruled out nor definitively assumed. Either way, the identification of a pattern characterised by metabolic markers indicating low energy supply in combination with reports of low social support and mental well-being underlines the importance of the interdisciplinary combination and consideration of factors in research on LPD and in practice. To promote the development of performance in athletes showing a pattern of low metabolism, social support and mental well-being, it is consequently advisable to broadly search for and address multiple possible causes. The Athlete Health and Readiness Checklist by Jeukendrup et al. [18], which contains an insufficient macronutrient supply as one of several addressable components, could be helpful here. Please note that this checklist is not validated and should therefore only be used with caution.

The present study's results also indicate the importance of psychosocial factors. More specifically, they give rise to speculation that for some cases of LPD the absence of social support might obstruct PD, as these athletes then profit from neither the emotional or instrumental aspects of social support, such as the use of appropriate coping mechanisms when dealing with CLEs [7, 25, 66] nor from being trained by supportive coaches [7, 24, 26]. However, the cross-sectional design of this study does not permit causal statements here.

Interestingly, this study's pre-analysis showed that social support and mental well-being are uncorrelated among elite athletes with LPD. Moreover, this can be seen in the identified patterns of the six-cluster solution revealing low levels in either both social support and mental well-being (subgroup three) or social support but not mental well-being (subgroup four) or vice versa (subgroups two and five). The following can be derived from this diverse picture: First, differentiating is important when investigating social support and mental well-being in the context of LPD. Especially the identification of patterns characterised by high or average social support and concomitant low mental well-being limit the general validity of previously reported positive associations between these psychosocial variables [27, 28, 59, 67]. Instead, it supports the assumption that a lack of social support is one of the several risk factors that can impair the mental well-being of elite athletes [22, 59]. Second, the failure to find a pattern characterised solely by low mental well-being can be interpreted in line with the view from Henriksen and Schinke [22] that mental well-being is an important resource for a good, long-term performance: Accordingly, mental well-being can become relevant for LPD when another resource is additionally weakened, which is indeed the case for two subgroups who have below-average values on, for example, the dimensions of metabolism or lower body dynamics. Notably, no resource-consuming factor becomes apparent at the group level for the subgroup with high social support but low mental well-being (subgroup five), suggesting at first glance that the explanation of weakened resources does not apply for them. Yet, there is a high statistical range within other variables such as CLEs, the inflammatory balance or the macro- and micronutrient supply. Considering previous literature on the role of burdening CLEs [25, 66], of inflammation and its balance [12, 68–70] or of macro- and micronutrient supply [14, 16, 22] for performance and its development, it is plausible that specifically at an individual level, one or several of these resources—but not social support—are strained or weakened. However, the causality of this statement must be examined in further research, because the study design does not allow causal conclusions. Nevertheless, we consider seeking a sport psychologist as a profitable first step to enhance PD when low social support or low mental well-being are group or individual characteristics. Such a professional can be an important, confidential source for fostering emotional or instrumental social support [71, 72]. Moreover, such contact can be important for assisting and—if needed—for collaborating with psychotherapists in clinical cases [19, 21, 73].

Finally, the identification of a subgroup without a characteristic pattern at group level (subgroup six) stresses the individual aspect of LPD. The low distinctiveness and stability of the cluster solutions moreover speaks for a high heterogeneity within the sample of elite athletes with LPD, which impedes generalisation. Assuming that there is always a reason for LPD at individual level, which is not necessarily visible at (sub-)group level, these results suggest that the ergodicity problem also applies to LPD. Although the observation that the individual level differs from the group level is not completely new to sport research (it previously has been demonstrated for athletic expertise [42] and the process of load and recovery [55]), this study underlines the importance of an individualised approach to enhance PD in most athletes. To enhance development in these cases, we hence recommend searching for and balancing potentially negative characteristics individually. Based on the current research on LPD (for a compilation, see Figure 1) and taking into account the variable range within this subgroup, the following variables might be worth considering: The insufficient supply with micronutrients such as vitamin D, vitamin B12, vitamin B9 and ferritin [14, 15] or with macronutrients and especially carbohydrates [16, 17, 74] not only impairs different body functions but might also impact negatively on performance. Notably, in subgroup six, there are several athletes who underscore the medical range for either ferritin or vitamin D, or whose carbohydrate and total caloric intake does not even reach 50% of the reference values recommended by DGE. Therefore, it might be advisable to address medical micronutrient and strong macronutrient deficiencies, sometimes even through supplementation [14]. Age and maturation are also candidates. It is known that athletic strength and speed decline from a certain age [75–77], and that in younger years, performance can be suppressed temporarily through, for instance, growth, maturation and related injuries [31, 62, 63]. Notably, this subgroup includes both the oldest and the youngest athlete, suggesting that it might be fruitful to consider and deal with possible limiting aspects of maturation or ageing by, for example, adapting training and focussing on strengthening, movement quality and skill maintenance [31, 62, 77]. Finally, the inflammatory imbalance is a candidate worth considering. Previously, an imbalance between pro- and anti-inflammatory responses was associated with low performance and its development [68, 78, 79]. Notably, in some members of subgroup six, the inflammatory imbalance far exceeds the average range for this study and comparable data from other research [79–81]. In these cases, we therefore recommend identifying and addressing possible causes of inflammatory balance that can range from exercise stress with insufficient recovery [69] to unrecognised oral inflammation [82].

## 5. Limitations and Strengths

In this study, the definition of LPD was based on subjective assessments by the respective national coaches. This might be a limitation due to bias [41, 83, 84]. However, coaches' assessments are generally considered to have a good prognostic validity, not least because their extensive implicit and explicit knowledge about the athlete is processed holistically [39–41]. Therefore, we consider the coaches' assessments to be a convenient and appropriate operationalisation of PD for this cross-sectional study design with benefits that overall outweigh possible limitations.

Distinctive for this study is its field-oriented, cross-disciplinary clustering design. This approach, however, entails following main limitations: First, being cross-sectional, this study's design does not permit causal statements. Second, the field-orientated approach required certain trade-offs between scientific standards and practical application in a high-performance setting. For instance, the estimation of energy requirements based on a uniform PAL-value is a standardised, economic method yet possibly oversimplifying the diverse physical activity levels across the cohort. Also, restricting the athletes only in terms of training on the day of measurement minimises the interference of the measurement with the training and daily routines, but accepts an influence of the inflammatory and physiological data through acute bouts of exercise the days prior to or by food intake at the measurement day [85, 86]. Here, the validity of the metabolic score in particular could have been enhanced by including additional metabolic markers, which are less sensitive to immediate dietary influences, such as estradiol or testosterone [16, 65]. Lastly, using CAs harbours the possibility that results are influenced by the cluster solution chosen or the variables selected for clustering [51]. The six-cluster solution, for instance, resulted in partly very small subgroups (*n* ≥ 5). While this cluster solution was selected to minimise the heterogeneity within a subgroup, this fine division appears to overfit the sample, suggesting limited generalisability. For these reasons, the three-cluster solution was also briefly outlined in this study. Given the instability of cluster solutions, the fundamental question arises as to the relevance of person-centred clustering in the context of LPD. These methods are currently highly valued in sports science, and it is hoped that they can contribute to the individualisation of training processes. However, despite the considerable amount of multidisciplinary data included in this study, it is uncertain how stable and generalisable the findings will ultimately be. The results furthermore might have been shaped by the decision to use the psychosocial variables of mental well-being and social support at the expense of stress-related factors for clustering. However, using stress-related factors would have led to methodological problems. Because stress is related to mental well-being [20, 21, 59], metabolism [86, 87] and inflammation [69, 86], the statistical requirement of independent variables for clustering would no longer be met.

Besides these limitations, the present study nonetheless stands out by considering information from several research areas in an exploratory CA. For example, the resulting simplification of the complex topic of PD facilitates data management, intervention planning and communication in research and practice [32, 33]. More importantly, the chosen study design also complied with the frequent demand for an unbiased and more comprehensive view on low performance and its development [11, 13, 18], and therefore can be considered as pioneering for this research context. Moreover, precisely because of the methodological aspects of the chosen design, this study opens avenues for future research questions such as how specific factors relate to individual PD over time.

## 6. Conclusion

In summary, results indicate that athletes with LPD can be clustered on the basis of similar underlying patterns of physiological, psychosocial and performance-related factors. Although the identified subgroups had domain-specific (low lower body dynamics; low social support), interdisciplinary combined (low lower body dynamics; low mental well-being; low metabolism; low social support) or indicatively no characteristics at subgroup level, patterns were not clearly distinguishable. This again emphasises the importance of an individualised approach to LPD in research and practice. Though the results should not be interpreted causally, this study's cross-disciplinary and multifactorial design breaks new ground in research on LPD in elite athletes and thus provides an important foundation for future investigations.

## Figures and Tables

**Table 1:** Overview of the variables used in this study.

	Variables used for clustering	Variables additionally used for cluster description
Demographic		Age, years of participation, sex

Anthropometric		Body mass index

Performance-related	Jump and sprint/tapping performance (*z* values averaged and renamed as lower body dynamics)	Acute injury, assessment of performance level

Psychosocial	PHQ-4 (score inverted and renamed as mental well-being)	Critical life events, PSS-4
	MSPSS (renamed as social support)	

Physiological	Ratio TNF-α:IL-10 (score inverted and renamed as inflammatory balance)	IFN-γ, IL-1β, IL-6, CRP, N:L ratio, IGFBP-1
	fT3, insulin, leptin (*z* values averaged and renamed as metabolism)	Ferritin, vitamin D, vitamin B12, vitamin B9

Menstrual or hormonal cycle		Prevalence of ir-/regular natural cycle and use of hormonal contraceptives, symptoms during cycle

Nutritional and sleep-related		Relative carbohydrate intake, relative total caloric intake, sleep duration, sleep quality

*Note:* fT3 = free triiodothyronine, N:L ratio = neutrophils-to-leucocytes ratio.

Abbreviations: CRP = C-reactive protein, MSPSS = multidimensional scale of perceived social support, PHQ-4 = patient health questionnaire 4, PSS-4 = perceived stress scale 4.

**Table 2:** Participant characteristics of the 62 elite athletes with LPD.

	Male	Female
Sex (*n*)	19	43
Discipline (*n*)		
Artistic gymnastics	0	11
3 × 3 basketball	0	3
Ice hockey	4	6
Modern pentathlon	5	1
Rhythmic gymnastics	0	5
Trampoline gymnastics	2	4
Table Tennis	1	1
Volleyball	7	12
Age (*M* ± SD; in years)	20.43 ± 4.79	18.66 ± 5.42
Years of practice (*M* ± SD)	12.25 ± 5.74	12.90 ± 5.34
Performance development (*M* ± SD)	3.89 ± 0.46	3.16 ± 0.84
Performance level (*M* ± SD)	4.32 ± 1.16	3.86 ± 1.19
Lower-body dynamics (*M* ± SD)		
Counter movement jump (in cm)	38.91 ± 6.25	30.54 ± 4.56
Drop jump (in cm)	28.52 ± 3.35	24.37 ± 4.02
Tapping (in Hz)	12.11 ± 1.39	10.79 ± 1.25
10 m Sprint (in s)	1.85 ± 0.13	1.91 ± 0.15
Psychosocial parameters (*M* ± SD)		
Mental well-being	4.92 ± 0.90	4.39 ± 1.08
Social support	4.55 ± 0.44	4.58 ± 0.43
Perceived stress	2.28 ± 0.84	2.43 ± 0.76
Critical life events	1.26 ± 2.37	1.86 ± 2.16
Physiological parameters (*M* ± SD)		
TNF-α (in pg/mL)	9.43 ± 8.99	7.08 ± 5.11
IL-10 (in pg/mL)	4.30 ± 4.92	3.16 ± 2.32
Ratio TNF-α:IL-10	3.22 ± 3.29	2.98 ± 1.88
Insulin (in pg/mL)	437.25 ± 319.48	614.38 ± 674.06
Leptin (in pg/mL)	655.82 ± 510.39	2965.42 ± 3533.64
fT3 (in pmol/L)	5.56 ± 0.57	5.06 ± 0.92

*Note:* The number of athletes (male, female, total) differed by discipline, precluding comparisons between disciplines as well as any statements regarding the relative frequency of LPD. Details of the performance in the sergeant jumps are not shown due to a small group size.

**Table 3:** Pattern characteristics (cluster centre, in brackets: minimum and maximum) for the identified subgroups.

Subgroup	1	2	3	4	5	6
*n*	5	5	3; all female	7	9; all female	33

Metabolism	*z* = 0.20 ± 0.67 (−0.39, 1.24)	*z* = −0.31 ± 0.45 (−0.77, 0.24)	***z* = −0.66 **±** 0.63 (−1.36, −0.14)**	*z* = −0.19 ± 0.38 (−0.70, 0.4)	*z* = 0.26 ± 0.44 (−0.38, 0.76)	*z* = −0.04 ± 0.44 (−0.99, 1.11)

Inflammatory balance	*z* = 0.23 ± 0.27 (−0.44, 0.24)	*z* = 0.13 ± 0.24 (−0.38, 0.27)		*z* = 0.17 ± 0.15 (−0.40, −0.03)	*z* = 0.04 ± 0.27 (−0.37, 0.55)	*z* = 0.12 ± 0.39 (−0.46, 1.62)

Lower-body dynamics	***z* = −1.64 **±** 0.81 (−2.72, −0.98)**	***z* = −0.87 **±** 0.43 (−1.51, −0.46)**		*z* = −0.46 ± 0.56 (−1.49, 0.10)	*z* = 0.36 ± 0.66 (−0.43, 1.26)	*z* = −0.03 ± 0.51 (−0.74, 1.06)

Social support	*z* = 0.21 ± 0.60 (−0.50, 1.09)	*z* = −0.00 ± 0.69 (−0.67, 0.92)	***z = *−0.63 **±** 0.89 (−1.57, 0.19)**	***z* = −1.59 **±** 0.84 (−2.81, −0.67)**	***z* = 0.54 **±** 0.64 (−0.50, 1.09)**	*z* = 0.56 ± 0.51 (−0.50, 1.09)

Mental well-being	*z* = 0.39 ± 0.48 (−0.09, 0.88)	***z = *−1.45 **±** 0.63 (−2.03, −0.57)**	***z* = −2.68 **±** 0.56 (−3.00, −2.03)**	*z* = −0.16 ± 0.71 (−1.55, 0.39)	***z = *−0.74 **±** 0.42 (−1.55, −0.09)**	*z* = 0.57 ± 0.47 (0.09, 1.36)

Further subgroup features	• Highest performance level • Least count of CLEs	• Highest percentage of acute injuries (80%) • Lowest levels of single inflammatory markers (e.g., IFN-γ, IL-1β, IL-6) as well as lowest average N:L ratio • Highest count of CLEs	• Lowest performance level • Lowest BMI • Highest levels of metabolic and single inflammatory markers (e.g., fT3, insulin, IFN-γ) • Lowest levels of vitamin B12 and vitamin D • Highest perceived stress • Most symptoms during menstrual cycle	• Highest proportion of female athletes with a regular (75.0%) and lowest proportion with an irregular (0.0%), natural menstrual cycle		• Oldest subgroup with most years of practice and high individual range • Lowest levels of perceived stress with high individual range • Fewest symptoms during menstrual cycle

*Note:* Characteristics of patterning relevance (indicated by cluster centres outside the average range) are highlighted by bold print. fT3 = free triiodothyronine, IFN-γ = interferon gamma, IL-1β = interleukin 1 beta, IL-6 = interleukin 6, N:L ratio =  neutrophils-to-lymphocytes ratio.

Abbreviations: BMI = body mass index, CLEs = critical life events, IGFBP-1 = insulin-like growth factor binding protein 1, REDs = relative energy deficiency in sports.

## Data Availability

The data that support the findings of this study are available upon request from the corresponding author. The data are not publicly available due to privacy or ethical restrictions.

## Supporting Information

The supporting information encompasses the dendrogram of the hierarchical CA with possible decision options for the number of subgroups (Supporting Figure A). Non-parametric correlations between performance-related, psychosocial, physiological, demographic, anthropometric, nutritional and sleep-related variables are presented in Supporting Table A. Descriptives and levels of significance for group comparisons between the identified subgroups for selected variables can be found in Supporting Table B.
