# Supplementary material for: Multifactorial Patterns of Low Performance Development in German Elite Athletes
Source: Transl Sports Med. 2025 Oct 5;2025:8421509. doi: 10.1155/tsm2/8421509 (PMC12515568; doi:10.1155/tsm2/8421509)
Supplement: Supporting Information — Additional supporting information can be found online in the Supporting Information section. [file 8421509.f1.docx]

**Supplementary Figure A:** Histogram inclusive normal curve and descriptives for the performance development across the 296 elite athletes.


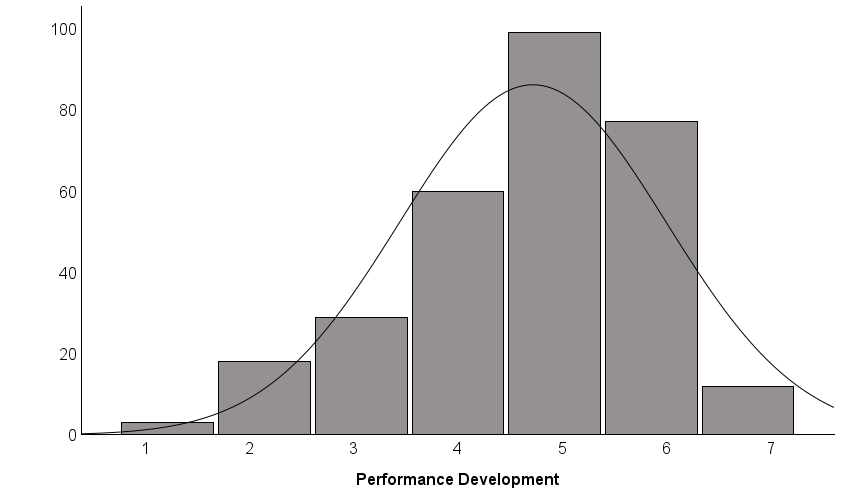


*M = 4.72, SD = 1.28; Median = 5.00*

Shapiro-Wilk:

*W* = 0.91, *p* < .001

1.0

2.0

6.0

7.0

**Performance Development**

0.0

20.0

40.0

60.0

80.0

100.0

3.0

4.0

5.0

**Supplementary Figure B:** Dendrogram of the hierarchical cluster analysis (Wards procedure).

Note: The final (solid line) and the two alternative decision options (dashed lines) for the number of subgroups are marked.


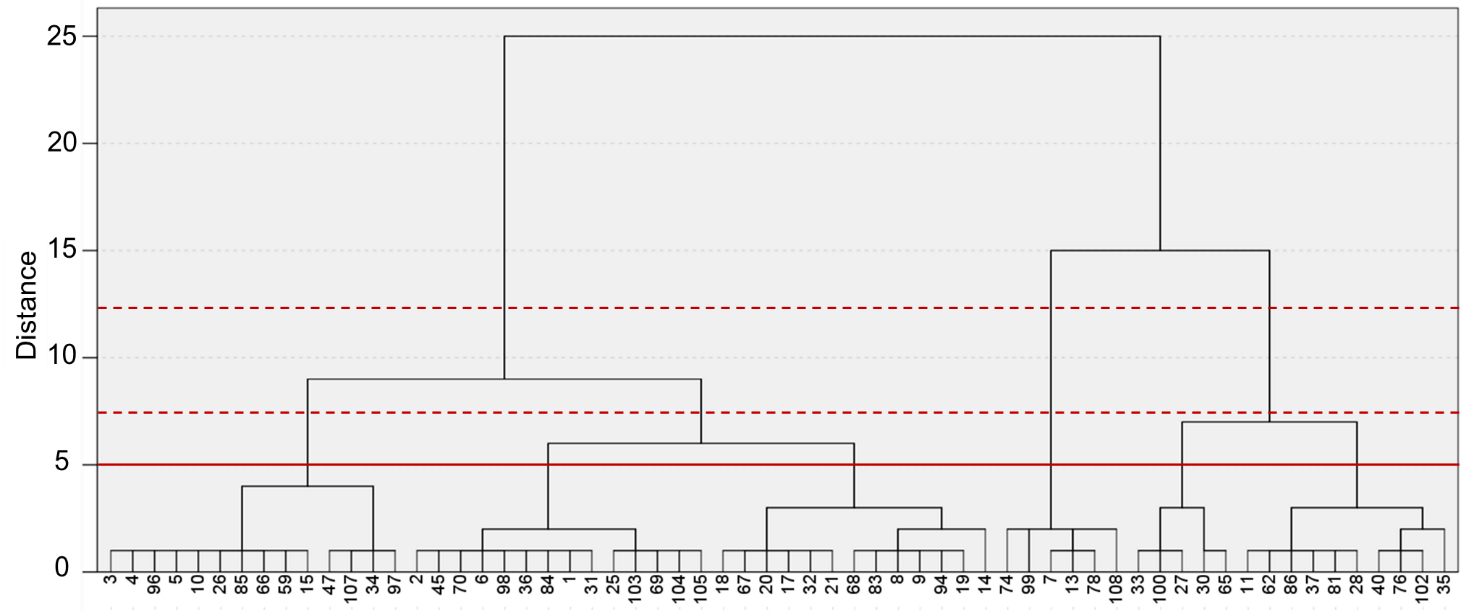


**Supplementary Table A:** Non-parametric correlations (Spearman’s Rho, in brackets: n) between performance-related, psychosocial, and physiological variables, as well as with demographic, anthropometric, nutritional, and sleep-related variables.

|  | Lower-body Dynamics | MSPSS | PHQ-4 | CLEs | PSS-4 | Insulin | Leptin | fT3 | TNFα: IL-10 |
| --- | --- | --- | --- | --- | --- | --- | --- | --- | --- |
| MSPSS | .053 (89) |  |  |  |  |  |  |  |  |
| PHQ-4 | .116 (92) | -.183 (98) |  |  |  |  |  |  |  |
| CLEs | -.012 (100) | -.019 (99) | **.367** (102)** |  |  |  |  |  |  |
| PSS-4 | -.017 (93) | **-.261** (99)** | **.537** (102)** | **.242* (103)** |  |  |  |  |  |
| Insulin | .174 (71) | .045 (73) | .047 (75) | -.110 (80) | .210 (76) |  |  |  |  |
| Leptin | .051 (85) | .099 (85) | -.086 (87) | -.106 (94) | .043 (88) | **.283* (80)** |  |  |  |
| fT3 | .128 (83) | .034 (84) | -.132 (87) | -.121 (92) | .004 (88) | **.311** (79)** | -.027 (89) |  |  |
| TNFα:IL-10 | .159 (71) | .159 (73) | .079 (75) | .135 (80) | -.196 (76) | -.040 (80) | -.036 (80) | .013 (79) |  |
| N:L- ratio | .099 (73) | **-.353** (72)** | .121 (75) | -.061 (80) | .181 (76) | .067 (70) | -.016 (78) | -.073 (80) | -.191 (70) |
| IFNγ | -.196 (82) | -.068 (82) | -.132 (84) | .010 (91) | .014 (85) | .099 (78) | **.266* (91)** | .112 (87) | -.107 (78) |
| IL-1β | -.106 (85) | -.089 (84) | -.101 (86) | -.089 (93) | -.008 (87) | .172 (79) | **.225* (93)** | .093 (88) | **-.317** (79)** |
| IL-6 | -.086 (71) | -.071 (73) | -.172 (75) | -.067 (80) | .008 (76) | .088 (80) | **.252* (80)** | **.356** (79)** | -.165 (80) |
| CRP | -.122 (77) | -.101 (77) | .028 (81) | -.018 (85) | .011 (81) | -.170 (75) | **-.252* (82)** | **-.312** (85)** | -.038 (75) |
| IGFBP-1 | -.045 (68) | .015 (70) | -.011 (72) | .094 (77) | -.126 (73) | **-.452** (77)** | **-.280* (77)** | -.080 (76) | .106 (77) |
| Ferritin | -.042 (87) | .087 (8) | -.124 (91) | -.012 (96) | **-.285** (92)** | -.204 (80) | **-.327** (92)** | -.046 (92) | -.054 (80) |
| Vitamin B9 | .140 (83) | .163 (84) | .130 (87) | .127 (92) | .005 (88) | -.114 (79) | -.020 (89) | -.126 (92) | .**227* (79)** |
| Vitamin D | -.200 (83) | .212 (84) | .001 (87) | **.220* (92)** | -.090 (88) | **-.440** (79)** | .079 (89) | **-.270** (92)** | .179 (79) |

| **Supplementary Table A. Continued.** | | | | | |  |  |  |  |  |
| --- | --- | --- | --- | --- | --- | --- | --- | --- | --- | --- |
|  | Lower-body Dynamics | MSPSS | PHQ-4 | CLEs | PSS-4 | | Insulin | Leptin | fT3 | TNFα: IL-10 |
| Vitamin B12 | -.093 (78) | .115 (79) | -.046 (82) | .025 (87) | -.084 (83) | | -.221 (74) | -.056 (84) | .081 (87) | -.022 (74) |
| Age | .017 (100) | .010 (99) | -.046 (102) | **.203* (110)** | -.136 (103) | | **-.340** (80)** | **-.240* (94)** | **-.479** (92)** | -.188 (80) |
| Years of practice | -.054 (92) | .088 (99) | -.135 (101) | .**250* (102)** | -.083 (102) | | -.146 (76) | -.067 (88) | **-.243* (87)** | -.121 (76) |
| Performance level | - .114 (100) | .117 (99) | - .090 (102) | - .071 (110) | -.109 (103) | | - .078 (80) | .049 (94) | - .005 (92) | - .097 (80) |
| BMI | -.020 (93) | .101 (92) | -.026 (95) | .111 (103) | -.120 (40) | | -.016 (75) | **.264* (014)** | -.154 (85) | -.121 (76) |
| Rel. Carbs | -.192 (38) | .085 (39) | -.008 (41) | -.056 (42) | -.029 (42) | | .209 (39) | **-.320* (41)** | .029 (39) | -.075 (39) |
| Rel. Tot. Kcal | -.306 (38) | .066 (39) | .066 (41) | -.107 (42) | .149 (41) | | .272 (39) | -.098 (41) | -.054 (39) | -.199 (39) |
| Sleep Quality | -.123 (31) | -.143 (33) | .089 (34) | .038 (35) | .123 (34) | | .019 (33) | .173 (35) | -.172 (33) | -.195 (33) |
| Sleep Duration | -.114 (27) | -.129 (29) | -.184 (30) | **-.395* (30)** | **-.466** (30)** | | .241 (30) | .172 (30) | **.411* (30)** | .450 (30) |
| Symptoms Menstrual Cycle (№) | .037 (31) | .043 (31) | .347 (31) | .245 (31) | .339 (31) | | .212 (31) | -.038 (31) | -.086 (31) | .156 (31) |

*Note*: ** Correlation significant for *p* <.001; * Correlation significant for *p* < .05. CLEs: Critical Life Events; MSPSS: Multidimensional Scale of Perceived Social Support; PHQ-4: Patient Health Questionaire-4; PSS-4: Perceived Stress Scale 4; IGFBP-1: Insulin-like Growth Factor Binding Protein 1; CRP: C-Reactive Protein; Rel. Carbs.: Relative Carbohydrate Intake; Rel. Tot. Kcal: Relative Total Caloric Intake.

| **Supplementary Table B:** Pattern characteristics (cluster centre, in brackets: minimum and maximum) according to the alternative, three- and four-cluster solution. | | | | | | | | | |
| --- | --- | --- | --- | --- | --- | --- | --- | --- | --- |
| **Three-cluster solution** | | | |  | | **Four-cluster solution** | | | |
| **Subgroup** | **1** | **2** | **3** | | **1** | | **2** | **3** | **4** |
| *n* | 25 | 12 | 25 | | 35 | | 7 | 10 | 10 |
| Metabolism | *z* = - 0.06 ± 0.48  (- 0.99, 1.24) | *z* = - 0.30 ± 0.50  (- 1.36, 0.41) | *z* = 0.09 ± 0.47 (- 0.68, 1.11) | | *z* = 0.01 ± 0.47 (- 0.99, 1.11) | | *z* = 0.18 ± 0.55 (- 0.39, 1.24) | *z* = - 0.21 ± 0.39 (- 0.77, 0.41) | *z* = -0.24 ±0.57 (- 1.36, 0.69) |
| Inflammatory Balance | *z* = - 0.14 ± 0.42 (- 0.46, 1.62) | *z* = - 0.18 ± 0.18 (- 0.40, 0.27) | *z* = - 0.09 ± 0.27 (- 0.41, 0.58) | | *z* = - 0.10 ± 0.38 (- 0.46, 1.62) | | *z* = - 0.26 ± 0.23 (- 0.44, 0.24) | *z* = - 0.15 ± 0.13 (- 0.40, - 0.01) | *z* = - 0.09 ± 0.30 (- 0.38,0.55) |
| Lower-Body Dynamics | ***z* = - 0.61 ± 0.69 (- 2.72, 0.60)** | *z* = - 0.51± 0.61 (- 1.51, 0.24) | *z* = 0.31 ± 0.53 (- 0.46, 1.26) | | *z* = 0.04 ± 0.51 (- 0.73, 1.06) | | ***z* = - 1.36 ± 0.81 (- 2.72, - 0.59**) | *z* = - 0.43 ± 0.57 (- 1.47, 0.33) | *z* = - 0.11 ± 0.46 (- 1.51, 1.26) |
| Social Support | *z* = 0.51 ± 0.64 (- 0.87, 1.09) | ***z* = - 1.03 ± 1.03 (- 2.81, 0.39)** | *z* = 0.44 ± 0.54 (- 0.50, 1.09) | | ***z* = 0.67 ± 0.46 (- 0.50, 1.09)** | | *z* = 0.16 ± 0.49 (- 0.50, 1.09) | ***z* = - 1.28 ± 0.85 (- 2.81, 0.92)** | *z* = - 0.04 ± 0.75 (- 1.57, 092) |
| Mental Well-being | ***z* = 0.66 ± 0.44 (- 1.36, 0.09)** | ***z* = - 1.35 ± 1.14 (- 0.39, 3.00)** | *z* = - 0.11 ± 0.68 (- 0.88, 1.55) | | *z* = 0.39 ± 0.56 (- 1.36, 0.58) | | ***z* = 0.60 ± 0.55 (- 1.36, 0.09)** | *z* = - 0.19 ± 0.63 (- 0.39, 1.55) | ***z* = - 1.84 ± 0.73 (1.06, 3.00)** |
| Further characteristics |  | Highest perceived stress |  | |  | |  |  |  |
| *Note:* Subgroup characteristics outside average range are marked by bold print. Further characteristics are mentioned if for the group-comparisons *p* ≤ .05. | | | | | | | | | |

| **Supplementary Table C:** Descriptives (M ± SD, in brackets: Minimum and Maximum) for demographic, anthropometric, performance related, sociopsychological and physiological variables plus levels of significance for group comparisons between the identified subgroups. | | | | | | | |
| --- | --- | --- | --- | --- | --- | --- | --- |
|  | Subgroup 1 | Subgroup 2 | Subgroup 3 | Subgroup 4 | Subgroup 5 | Subgroup 6 | Sig. |
| sex | *n*_male_ = 3  *n*_female_ = 2 | *n*_male_ = 1  *n*_female_ = 4 | *n*_male_ = 0  *n*_female_ = 3 | *n*_male_ = 3  *n*_female_ = 4 | *n*_male_ = 0  *n*_female_ = 9 | *n*_male_ = 12  *n*_female_ = 21 | *p* = .126 |
| Injured athletes | 20.0% | 80.0% | 66.7% | 14.3% | 44.4% | 30.3% | *p* = .167 |
| Age (in years) | 17.77 ± 5.39 (13.58, 27.09) | 17.52 ± 1.44 (15.75, 18.92) | 17.19 ± 2.01 (14.91, 18.75) | 20.19 ± 3.66 (14.66, 24.66) | 16.74 ± 2.63 (13.42, 20.50) | 20.31 ± 6.33 (13.42, 40.17) | *p* = .452 |
| Years of practice | 10.03 ± 4.51 (5.25, 16.50) | 11.50 ± 2.22 (8.25, 14.08) | 9.67 ± 3.93  (5.92, 13.75) | 12.25 ± 3.33 (8.58, 17.42) | 11.60 ± 2.28 (9.00, 14.58) | 13.96 ± 6.67 (4.92, 33.75) | *p* = .680 |
| Performance level | 4.20 ± 0.84  (3, 5) | 3.80 ± 0.84  (3, 5) | 3.67 ± 1.15  (3, 5) | 4.00 ± 1.41  (2, 6) | 3.78 ± 0.83  (3, 5) | 4.09 ± 1.35  (1, 7) | *p* = .926 |
| BMI (in kg/m²) | 21.17 ± 2.52 (19.12, 24.47) | 22.01 ± 2.85 (19.21, 26.69) | 19.35 ± 2.35 (16.96, 21.66) | 22.42 ± 1.62 (20.81, 24.46) | 20.53 ± 2.58 (15.20, 23.54) | 22.23 ± 2.79 (17.57, 27.29) | *p* = .449 |
| Critical Life Events (count) | 0.40 ± 0.55  (0.00, 1.00) | 3.40 ± 2.70  (1.00, 8.00) | 3.33 ± 3.51  (0.00, 7.00) | 2.57 ± 3.91  (0.00, 10.00) | 2.22 ± 2.28  (0.00, 7.00) | 1.12 ± 1.41 (0.00, 5.00) | *p* = .097 |
| Perceived Stress (arbitraty units) | 2.30 ± 0.91  (1.00, 3.25) | 2.85 ± 0.51  (2.25, 3.50) | 3.33 ± 0.72  (2.50, 3.75) | 2.82 ± 0.70  (1.50, 3.50) | 2.62 ± 0.75  (1.33, 3.75) | 2.08 ± 0.71  (1.00, 4.00) | *p* = .015 |
| Cycle information (proportion among female) | INC: 100.0% | RNC: 50.0%  INC: 50.0% | INC: 66.7%  HC: 33.3% | RNC: 75.0%  HC: 25.0% | RNC: 50.0%  INC: 37.5%  HC: 12.5% | PM: 15.0%  RNC: 30.0%  INC: 25.0%  HC: 30.0% | *p* = .417 |

| **Supplementary Table C. Continued.** | | | | | | | |
| --- | --- | --- | --- | --- | --- | --- | --- |
|  | Subgroup 1 | Subgroup 2 | Subgroup 3 | Subgroup 4 | Subgroup 5 | Subgroup 6 | Sig. |
| Symptoms Menstrual Cycle (count) | 1  (1) | 3.67 ± 3.79  (1, 8) | 5.50 ± 3.54  (3, 8) | 2.00 ± 1.83  (0, 4) | 2.43 ± 2.07  (0, 5) | 1.71 ± 1.73  (0, 6) | *p* = .648 |
| IFN-γ (in ng/ml) | 0.48 ± 0.99  (0.01, 2.25) | 0.06 ± 0.03  (0.01, 0.08) | 1.66 ± 2.86  (0.00, 4.97) | 0.08 ± 0.11  (0.01, 0.31) | 0.09 ± 0.18  (0.00, 0.52) | 0.45 ± 1.06 (0.00, 4.32) | *p* = .996 |
| IL-1β (in pg/ml) | 23.60 ± 15.48 (2.21, 42.99) | 8.06 ± 6.78  (3.14, 19.21) | 12.57 ± 15.84 (1.98, 30.78) | 16.18 ± 24.54 (1.45, 67.17) | 16.16 ± 14.33 (0.27, 41.11) | 16.24 ± 22.97 (0.12, 86.30) | *p* = .769 |
| IL-6 (in pg/ml) | 10.41 ± 12.64 (1.87, 31.54) | 2.48 ± 1.19  (1.00, 3.82) | 9.53 ± 15.48 (0.41, 27.41) | 4.19 ± 7.93  (0.42, 22.08) | 2.96 ± 2.97  (0.53, 9.90) | 4.48 ± 5.62 (0.67, 29.45) | *p* = .375 |
| IL-10 (in pg/ml) | 4.70 ± 2.77  (1.31, 7.66) | 2.91 ± 1.88  (0.88, 5.88) | 4.62 ± 5.17  (0.82, 10.52) | 2.53 ± 1.89  (0.94, 5.59) | 2.71 ± 1.96  (0.45, 5.70) | 3.74 ± 3.96 (0.45, 19.64) | *p* = .709 |
| TNF-α (in pg/ml) | 7.45 ±3.25  (4.94, 13.02) | 6.59 ±1.08  (5.21, 8.04) | 12.39 ±16.26 (1.77, 31.12) | 5.31 ±1.63  (3.32, 8.55) | 7.93 ± 5.03  (3.21, 17.06) | 8.12 ±7.31 (2.32, 30.94) | *p* = .709 |
| CRP (in mg/dl) | 0.05 ± 0.09  (0.00, 0.18) | 0.14 ± 0.24  (0.01, 0.57) | 0.06 ± 0.03  (0.04, 0.09) | 0.10 ± 0.11  (0.00, 0.25) | 0.03 ± 0.03  (0.00, 0.10) | 0.07 ± 0.07 (0.00, 0.32) | *p* = .347 |
| IGFBP-1  (in ng/ml) | 8.99 ± 6.36  (2.74, 19.23) | 33.59 ± 25.15 (6.99, 62.15) | 41.94 ± 34.98 (17.20, 66.67) | 22.56 ± 14.82 (7.09, 44.60) | 20.88 ± 28.43 (2.04, 83.68) | 23.57 ± 22.64 (1.87, 104.60) | *p* = .254 |
| Insulin (in ng/ml) | 0.64 ± 0.44  (0.15, 1.15) | 0.37 ± 0.31  (0.14, 0.92) | 0.29 ± 0.18  (0.12, 0.48) | 0.38 ± 0.27  (0.05, 1.11) | 1.03 ±1.16  (0.14, 3.15) | 0.53 ± 0.43  (0.11, 1.98) | *p* = .679 |
| Leptin (in ng/ml) | 4.77 ± 7.22  (0.53, 15.53) | 0.91±0.48  (0.43, 1.52) | 1.06 ± 1.10  (0.22, 2.31) | 1.81 ± 1.73  (0.29, 4.56) | 1.73, 0.88  (0.63, 3.26) | 2.56 ± 3.38 (0.18, 17.28) | *p* = .716 |

|  | Subgroup 1 | Subgroup 2 | Subgroup 3 | Subgroup 4 | Subgroup 5 | Subgroup 6 | Sig. |
| --- | --- | --- | --- | --- | --- | --- | --- |
| fT3 (in pmol/l) | 5.70 ± 0.69  (4.91, 6.48) | 4.90 ± 0.96  (3.99, 6.22) | 4.12 ± 1.46  (2.59, 5.51) | 5.06 ± 0.65  (4.15, 5.90) | 5.72 ± 0.76  (4.52, 6.99) | 5.19 ± 0.79 (3.47, 6.58) | *p* = .235 |
| Ferritin (in ng/ml) | 52.00 ± 36.93 (14.40, 99.70) | 24.62 ± 21.78 (9.30, 62.40) | 34.97 ± 20.20 (20.80, 58.10) | 43.41 ± 17.82 (22.90, 65.00) | 30.18 ± 18.25 (9.40, 65.30) | 55.26 ± 38.46 (12.00, 172.00) | *p* = .129 |
| Vitamin B9  (in ng/ml) | 9.36 ± 7.42  (4.48, 20.20) | 9.82 ± 2.23  (6.70, 12.30) | 12.25 ± 5.00 (8.94, 18.00) | 8.10 ± 4.67  (2.02, 16.30) | 9.02 ± 4.55  (4.51, 18.70) | 8.96 ± 5.31 (4.40, 32.60) | *p* = .483 |
| Vitamin B12  (in ng/ml) | 0.56 ± 0.21  (0.32, 0.75) | 0.48 ± 0.22  (0.17, 0.71) | 0.39 ± 0.31  (0.16, 0.74) | 0.43 ± 0.23  (0.17, 0.87) | 0.45 ± 0.10  (0.34, 0.60) | 0.45 ± 0.19 (0.22, 1.02) | *p* = .783 |
| Vitamin D (in ng/ml) | 30.18 ± 12.79 (19.90, 47.90) | 31.90 ± 12.54 (15.50, 47.30) | 22.30 ± 4.36 (18.60, 27.10) | 30.40 ± 12.19 (19.30, 52.20) | 26.27 ± 10.97 (17.50, 53.80) | 27.47 ± 10.83 (12.80, 54.30) | *p* = .800 |
| Relative Carbo-hydrate Intake (in %) | 66.00 ± 5.66 (62.00, 70.00) | 66.33 ± 12.10 (57.00, 80.00) |  |  | 58.40 ± 21.78 (44.00, 97.00) | 63.22 ± 16.19 (32.00, 93.00) | *p* = .694 |
| Relative Protein Intake (in %) | 73.00 ± 33.94 (49.00, 97.00) | 67.00 ± 25.12 (52.00, 96.00) |  |  | 86.80 ± 38.47 (35.00, 137.00) | 66.00 ± 23.37 (29.00, 108.00) | *p* = .621 |
| Relative Fat Intake  (in %) | 183.00 ± 16.97 (171.00, 195.00) | 142.00 ± 46.12 (111.00, 195.00) |  |  | 210.00 ± 154.47 (90.00, 465.00) | 149.00 ± 49.77 (73.00, 265.00) | *p* = .626 |
| Relative Total Caloric Intake (in %) | 73.50 ± 12.02 (65.00, 82.00) | 69.67 ± 18.50 (58.00, 91.00) |  |  | 73.20 ± 30.63 (43.00, 123.00) | 67.83 ± 16.57 (40.00, 105.00) | *p* = .871 |
| Sleep Duration  (in hours) | 8.47 ± 0.76  (7.93, 9.00) | 8.28 ± 0.09  (8.17, 8.33) |  |  | 8.39 ± 1.26  (6.67, 10.17) | 8.38 ± 0.86 (7.17, 10.00) | *p* = .991 |
| Sleep Quality  (arbitrary units) | 2.67 ± 0.47  (2.33, 3.00) | 2.67 ± 0.34  (2.33, 3.00) |  |  | 2.37 ± 0.73  (1.50, 3.00) | 2.61 ± 0.83 (1.33, 4.00) | *p* = .907 |

**Supplementary Table C. Continued.**

*Note:* Sig.: Significance; BMI: body-mass-index; RNC: regular, natural menstrual cycle; INC: irregular, natural menstrual cycle; HC: hormonal contraceptives; PM: premenstrual; IGFBP-1: Insulin-like Growth Factor Binding Protein 1; CRP: C-Reactive Protein; For the group comparisons, the Chi²-test was used for age and gender, and the Kruskal-Wallis-test for all other variables. The performance level was assessed by the respective national trainer (0: very low – 7: very high).
